# Smartphone Sensor-Based Human Motion Characterization with Neural Stochastic Differential Equations and Transformer Model

**DOI:** 10.3390/s22197480

**Published:** 2022-10-02

**Authors:** Juwon Lee, Taehwan Kim, Jeongho Park, Jooyoung Park

**Affiliations:** Department of Control and Instrumentation Engineering, Korea University, 2511 Sejong-ro, Sejong-City 30019, Korea

**Keywords:** smartphone sensors, human motion, deep learning, neural stochastic differential equations, transformer, GPT2

## Abstract

With many conveniences afforded by advances in smartphone technology, developing advanced data analysis methods for health-related information from smartphone users has become a fast-growing research topic in the healthcare field. Along these lines, this paper addresses smartphone sensor-based characterization of human motions with neural stochastic differential equations (NSDEs) and a Transformer model. NSDEs and modeling via Transformer networks are two of the most prominent deep learning-based modeling approaches, with significant performance yields in many applications. For the problem of modeling dynamical features, stochastic differential equations and deep neural networks are frequently used paradigms in science and engineering, respectively. Combining these two paradigms in one unified framework has drawn significant interest in the deep learning community, and NSDEs are among the leading technologies for combining these efforts. The use of attention has also become a widely adopted strategy in many deep learning applications, and a Transformer is a deep learning model that uses the mechanism of self-attention. This concept of a self-attention based Transformer was originally introduced for tasks of natural language processing (NLP), and due to its excellent performance and versatility, the scope of its applications is rapidly expanding. By utilizing the techniques of neural stochastic differential equations and a Transformer model along with data obtained from smartphone sensors, we present a deep learning method capable of efficiently characterizing human motions. For characterizing human motions, we encode the high-dimensional sequential data from smartphone sensors into latent variables in a low-dimensional latent space. The concept of the latent variable is particularly useful because it can not only carry condensed information concerning motion data, but also learn their low-dimensional representations. More precisely, we use neural stochastic differential equations for modeling transitions of human motion in a latent space, and rely on a Generative Pre-trained Transformer 2 (GPT2)-based Transformer model for approximating the intractable posterior of conditional latent variables. Our experiments show that the proposed method can yield promising results for the problem of characterizing human motion patterns and some related tasks including user identification.

## 1. Introduction

Due to advancements in smartphone technology, a variety of sensors have become available for smartphones, with accompanying applications to process information obtained from embedded sensors. Modern sensors such as inertial measurement unit (IMU) sensors in smartphones allow for more advanced data analysis and studies of user data. In this paper, we intend to deal with healthcare-related data analysis problems utilizing smartphone sensors. More specifically, we consider the problem of characterizing the human movements of walking, running, squats, and jumping jacks by means of a modern deep learning approach. Research on analyzing, characterizing, and recognizing human motion has been conducted by many researchers in the healthcare-related fields [1,2,3,4,5,6,7,8,9,10,11,12,13]. In particular, machine learning [1,2,13] and deep learning [3,4,5,6,7,8,9,10,11,12] methods have been widely applied to the analysis of human motion pattern and the recognition of activities. Wang et al. [1] proposed the use of a Gaussian process for constructing dynamical models to characterize human motion from captured motion data. It is capable of considering both dynamical and observational mappings with small datasets. For analyzing human motion, Kim et al. [2] proposed a model which consists of a variational autoencoder and a Gaussian process for characterizing motion dynamics in latent space and novelty (e.g., fall) detection. It treats noisy high-dimensional raw data as a low-dimensional latent feature, which represents characteristics of human dynamic walking data well. However, the work of Ref. [2] depends on multiple sensors (e.g., smartphones on the wrist and leg), and the Gaussian process-based model is less widely used compared to that of typical deep neural networks. Kim et al. [3] analyzed IMU sensor data with two-stage latent dynamics modeling and filtering (TS-LDMF), consisting of deep learning-based latent space modeling and nonlinear filtering. It has the advantage of representing smooth trajectories on low-dimensional latent space well for noisy sensor data (e.g., walking and running). However, the first-stage of TS-LDMF relies on future observations to form the approximate posterior distribution for latent variables. Uddin et al. [4] considered human activity recognition based on a Long Short-Term Memory (LSTM) based model, referred to as Neural Structured Learning (NSL), which distinguished among different human activities, including walking. The proposed models show better performance than the typical deep learning model (e.g., Recurrent Neural Network (RNN), Convolutional Neural Network (CNN), and Deep Belief Network (DBN)). For its excellent performance, this model utilized not only IMU sensor data, but model input also contained ECG (electrocardiogram) data, which is difficult to measure in daily life. Mukherjee et al. [5] proposed an ensemble model consisting of CNN-Net, Encoded-Net, and CNN-LSTM to categorize human motion data from sensors. In the ensemble, each model executes the role of classifier, and then the final prediction result is obtained by means of majority voting. Since the method of [5] uses an ensemble model of three different models, its performance is better than when using only one methodology. While its performance is good, it may be time-consuming due to the need to train and evaluate voting. Ronao et al. [6] adapted CNNs for human activity recognition (HAR) using smartphone sensor data, and considered activities consisting of six different motions. Here, the convolutional layer can extract valuable features without any pre-processing (e.g., feature selection and feature hand-crafting) from raw data. The work focused on the use of CNN for human activity recognition, whereas our concern is with the use of the more advanced Neural Stochastic Differential Equation (SDE) [14] and Transformer [15]. Jiang et al. [7] considered CNNs for recognizing human activity with IMU sensors data and activity images. Here, the method of [7] considered images through discrete Fourier Transform (DFT) as input to the model. By using the Deep Convolutional Neural Networks (DCNN), it can extract discriminative features for activity recognition. However, the work of [7] was only supported by some existing data from UCI, USC, and SHO. Wang et al. [8] proposed a motion recognition method with a multi-layer perceptron (MLP) network, which utilizes frequency domain features from a DFT. Here, the wavelet transform analysis, which enables selection and analysis of a meaningful frequency band, is also utilized for input to the model. It can offer additional information about motion signals. In spite of its advantages, the proposed method is applied for only walking data. Hence, performance cannot be guaranteed for other motions. Khan et al. [9] proposed a human activity recognition framework, which is an attention-based multi-head model. The model consists of three lightweight convolutional heads. Its feature extraction capability was strengthened by means of the attention-based model. However, according to their results, there exists room for further improvement in its capabilities for distinguishing some activities. Augustinov et al. [10] proposed the attention-based Transformer model for recognizing daily activities. The procedure is conducted on two levels. In the first level, the probability scores of activities are extracted, and then the Transformer-based model classifies the activities in the second level. Compared to LSTM networks, their proposed method outperforms the others. Despite its excellent performance, however, overfitting may occur during training. Shi et al. [11] considered human activity recognition with a residual multi-layer perceptron (Res-MLP), which contains linear layers and a Gaussian error linear unit (GELU). For classifying six activities, data from smartphone gyroscopes and accelerometers were utilized as inputs to the model. While it showed high performance for the UCI-HAR dataset from UCI, it required an extra procedure for filtering noise out of the raw data.

For the purpose of addressing smartphone sensor-based characterization of human motions, we use a deep learning approach based on neural stochastic differential equations [14] and a Transformer model [15]. Neural stochastic differential equations and modeling via Transformer networks are two of the most prominent deep learning-based modeling approaches, with significant performance yields in many applications. For the problem of modeling dynamical features, stochastic differential equations and deep neural networks are frequently used paradigms in science and engineering, respectively. Combining these two paradigms in one unified framework has drawn significant interest in the deep learning community, and neural stochastic differential equations [14] are among the leading technologies for combining these efforts. In this paper, the neural SDE plays the role of transition model in the generative component of the formulation. Recently, the use of attention has become a widely adopted strategy in many deep learning applications, and a Transformer [15] is a deep learning model that uses the mechanism of self-attention. This concept of a self-attention based Transformer was originally introduced for tasks of natural language processing (NLP), and due to its excellent performance and versatility, the scope of its applications is rapidly expanding. The structure of Transformer consists of an encoder block and a decoder block, which consist of a self-attention layer and a fully connected layer. The encoder block converts the input features into a latent representation, and the decoder block provides the outputs that meet the users’ desired purpose (e.g., prediction, classification, etc.). In this paper, our inference networks utilize a Transformer-based auto-regressive model called Generative Pre-trained Transformer 2 (GPT2) [16], which is a recently introduced enhanced auto-regressive version of the Transformer. GPT2 relies on the form of stacked decoder Transformer, which inputs a sequence of tokens and applies embeddings for position and token, and then is followed by several decoder layers. Each layer applies multi-head self-attention combined with a feedforward network, layer normalization, and residual connections. By utilizing the techniques of neural stochastic differential equations and a Transformer model along with data obtained from smartphone sensors, we present a deep learning method capable of efficiently characterizing human motions. For characterizing human motions, we encode the high-dimensional sequential data from smartphone sensors into latent variables in a low-dimensional latent space. The concept of the latent variable is particularly useful because it can not only carry condensed information concerning motion data, but also learn their low-dimensional representations.

The paper is organized as follows: In Section 1, we briefly explain the rationale for this paper, and introduce related works. In Section 2, we provide general concepts of neural stochastic differential equations and the GPT2 Transformer model as main tools for characterizing human motions based on smartphone sensor signals, and propose a modern variational inference approach for solving the characterization problem. In Section 3, after presenting the process for acquiring the data used in the experiments, we report on the applicability of the proposed approach to smartphone sensor-based characterization of human motions, and describe the results of the experiments. In the final Section 4, we provide our discussion and conclusions.

## 2. Methods

**Figure 1:**
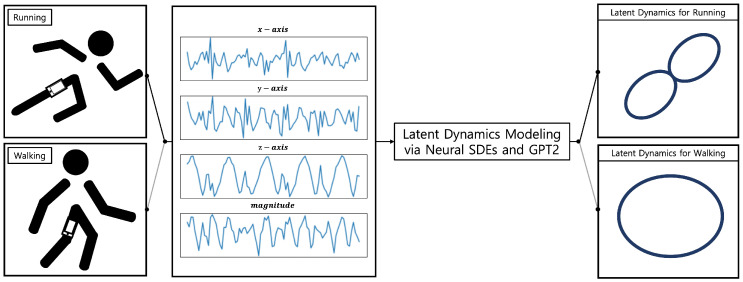
Neural stochastic differential equations and Transformer-based modeling.

As a framework for smartphone sensor-based characterization of human motions, we present a latent dynamical model incorporating neural SDEs [14] and GPT2 [16], which is an enhanced auto-regressive version of the Transformer. Our approach yields low-dimensional latent trajectories of human motions including walking and running by processing high-dimensional raw data from smartphone sensors, as shown in Figure 1. In the following, we derive the framework in a step-by-step manner after providing relevant preliminaries.

### 2.1. Preliminaries

#### 2.1.1. Neural Stochastic Differential Equations

A stochastic differential equation can be formally understood as a noise-driven ordinary differential equation in the sense that

(1) $$\frac{dz_{t}}{dt}=\mu \left(z_{t},t\right)+\sigma \left(z_{t},t\right)w_{t},$$

where the forcing function $w_{t}$ is a stochastic process often modeled as white noise [17]. Using neural networks for the $\mu (z_{t},t)$ and $\sigma (z_{t},t)$, one can construct so-called neural stochastic differential equations [14]. Thus, for a fixed final time $T_{f}>0$, one can describe a stochastic process $z_{t}$ that continuously evolves over time with the framework of neural SDEs. More specifically, neural SDEs are *k*-dimensional stochastic differential equations describing the stochastic dynamics of $z_{t}$ in the following form [14]:

(2) $$dz_{t}=\mu_{\theta }\left(z_{t},t\right)dt+\sigma_{\theta }\left(z_{t},t\right)dW_{t},0\leq t\leq T_{f},$$

where both $\mu_{\theta }$ and $\sigma_{\theta }$ are neural networks, and $W:\left[0,T_{f}\right]\to R^{n}$ is an *n*-dimensional standard Brownian motion. Note that, in the above neural SDEs, neural networks $\mu_{\theta }:R^{k}\times \left[0,T_{f}\right]\to R^{k}$ and $\sigma_{\theta }:R^{k}\times \left[0,T_{f}\right]\to R^{k\times n}$ are both collectively parameterized by θ because they belong to the generative component described by the parameter θ. Here, *k* is the dimension of latent state, and *n* is the number of noise sources. Throughout this paper, we consider the k=2 case for the convenience of visualization and characterization with latent trajectories. Extension to the k=3 case is straightforward, and when more dimensions are needed for k (i.e., k≥4), one can obtain an approximate visualization with the help of dimension reduction tools such as PCA [18] and incremental PCA [19]. Following the concept of the Ito integral (e.g.,  [17]), the solution of the neural SDEs can be represented as the continuous-time stochastic process $z_{t}$ that satisfies the integral equation

(3) $$z_{t}=z_{0}+\int_{0}^{t}\mu_{\theta }\left(z_{s},s\right)ds+\int_{0}^{t}\sigma_{\theta }\left(z_{s},s\right)dW_{s}$$

with an initial condition $z_{0}$. It is well known that neural stochastic differential equations can be utilized for modeling dynamics in a variety of contexts [14]. Finite-dimensional solutions to stochastic differential equations are rarely of closed-form [14], and need to be approximated in many practical problems. We approximate the solutions to neural stochastic differential equations using the Euler–Maruyama method [17].

#### 2.1.2. Transformer Model

**Figure 2:**
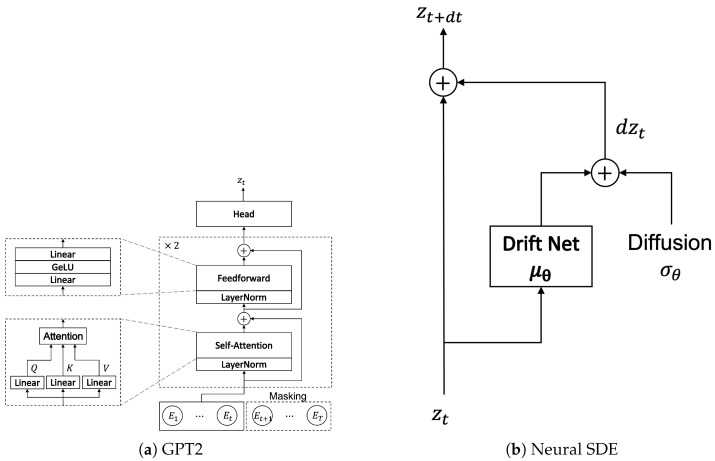
The GPT2 and Neural SDE architectures used in this study.

Transformer [15] was originally introduced as a machine learning solution to language translation tasks. Since its introduction as a novel solution to natural language processing, it has exhibited successful performance across a number of application domains, and is currently the network of choice for a majority of deep learning researchers. The main structure of Transformer consists of an encoder and a decoder. The encoder transforms a given sequence of input tokens into latent representations. The decoder generates an output sequence in an auto-regressive manner. Since it proceeds auto-regressively, the decoder takes all previously generated tokens as its own input at each step of an inference. The attention mechanism adopted in most Transformers is the scaled dot-product attention, which can quantify the correlation of input sequences. The scaled dot-product attention is defined as

(4) $$\mathrm{Attention}\left(Q,K,V\right)=\mathrm{softmax}\left(\frac{QK^{⊤}}{\sqrt{d_{K}}}\right)V,$$

where *Q*, *K*, *V* are vectors of the queries, keys, and values, respectively. *Q* and *K* have a common dimension, which is denoted by $d_{K}$. GPT2 [16] is a recently introduced variant of Transformer. It relies on the form of stacked decoder Transformer, which inputs a sequence of tokens and applies embeddings for position and token, and then is followed by several decoder layers. Each layer applies multi-head self-attention combined with a feedforward network, layer normalization, and residual connections. Here, in this study, we use a small GPT2 structure consisting of two layers and a single head, which turns out to be sufficient for our purpose. Since the main concern of this paper is smartphone or mobile applications, considering small sizes for the structure should suffice. The architecture of the GPT2 Transformer used in this study is shown in Figure 2a.

### 2.2. Problem Formulation and Training

#### 2.2.1. Generative Component: Neural SDEs and Decoder

In this subsection, we describe the generative component of the proposed framework, where the neural SDE plays a critical role. The generative component involves a transition network and a decoder network. The transition network represents a stochastic dynamical system for latent variables. For the transition network, we use a simplified neural SDE model of Figure 2b, which modifies the most general neural SDE model

(5) $$dz_{t}=\mu_{\theta }\left(z_{t},t\right)dt+\sigma_{\theta }\left(z_{t},t\right)dW_{t}$$

into the following form:

(6) $$dz_{t}=\mu_{\theta }\left(z_{t}\right)dt+\sigma_{\theta }dW_{t}.$$

This modification is for the sake of convenience in visualization and interpretation. In the simplified model, the drift term $\mu_{\theta }\left(\cdot \right)$ is a multi-layer perceptron (MLP) network [20] taking $z_{t}$ as its only input, and the diffusion network is replaced by a parameter indicating the noise magnitude $\sigma_{\theta }$. The exact structure of the drift MLP network is specified in the Appendix A. The decoder network is a measurement model for sensors (e.g., [21]), which represents the conditional distribution of observations given the latent values. Note that, here, our notation uses the parameter θ for all the parameters of the generative component, which includes the drift neural network, the diffusion term, and the decoder network.

For the decoder representation, one may have several choices, for which reconstruction (i.e., $p_{\theta }\left(x_{t}|z_{t}\right)$) [21,22] and prediction (i.e., $p_{\theta }\left(x_{t+1}|z_{t}\right)$) [23] are widely used. Training of the reconstruction decoder has the obvious interpretation of maximizing likelihood of observations. On the other hand, prediction is not only a powerful strategy for modern unsupervised learning [24,25], but also a powerful conventional technique in signal processing for compressing data. In this paper, we empirically found that the use of $p_{\theta }\left(x_{t}|z_{t}\right)$ or $p_{\theta }\left(x_{t+1}|z_{t}\right)$ for the decoder yielded too much oscillation or smoothness, respectively, in the resultant latent trajectories, and using their average (meaning $p_{\theta }\left(\left(x_{t}+x_{t+1}\right)/2|z_{t}\right)$) was just right for our purpose. For simplicity and convenience of notation, we write $(x_{t}+x_{t+1})/2$ as ${\bar{x}}_{t+1}$ throughout this paper. For the prior distribution of initial latent state, we use $p\left(z_{0}\right)=N\left(\mu_{0},\sigma_{0}^{2}I_{2}\right)$. We obtain the starting mean vector of the latent sequence, $\mu_{0}$, by performing a principal component analysis (PCA) with $x_{-m:0}$ as the inputs, where *m* is a small non-negative integer. In our experiments, we use m=0 for simplicity, and in this case, $\mu_{0}\left(z_{0}\right)$ is the PCA projection of $x_{0}$ onto the two-dimensional latent space. For the variance value of the prior distribution, we use $\sigma_{0}^{2}=0.2^{2}$.

Owing to the Markov property [21] of the latent dynamics, the joint probability distribution for the observations, ${\bar{x}}_{1:T+1}$, and the latent variables, $z_{0:T}$ can be factorized as follows:

(7) $$p_{\theta }\left({\bar{x}}_{1:T+1},z_{0:T}\right)=p\left(z_{0}\right)p_{\theta }\left({\bar{x}}_{1}|z_{0}\right)\prod_{t=1}^{T}p_{\theta }\left({\bar{x}}_{t+1}|z_{t}\right)p_{\theta }\left(z_{t}|z_{t-1}\right),$$

where $p(z_{0})$, $p_{\theta }\left({\bar{x}}_{t+1}|z_{t}\right)$, and $p_{\theta }\left(z_{t}|z_{t-1}\right)$ stand for the probability distribution of the initial latent variable, the conditional probability distribution for the decoder network, and the conditional probability distribution for the transition network, respectively. Note that the probabilistic model of Equation (7) is based on the key idea that the sequence of the high-dimensional sequential observation, ${\bar{x}}_{1:T+1}$, can be explained by means of the lower-dimensional sequence of the latent variables, $z_{0:T}$, where the $z_{0:T}$ are generated via the conditional distribution of the transition network, $p_{\theta }\left(z_{t}|z_{t-1}\right)$, and the ${\bar{x}}_{1:T+1}$ are generated via the conditional distribution of the decoder network, $p_{\theta }\left({\bar{x}}_{t+1}|z_{t}\right)$. In this paper, the decoder network is a multi-layer perceptron [20], the structure of which is provided in the Appendix A. We will describe in greater detail how our problem as formulated can be solved by variational inference [26].

#### 2.2.2. Variational Distributions

One can obtain the following factorization for the posterior $p_{\theta }\left(z_{0:T}|x_{1:T+1}\right)$ based on the previous factorization in Equation (7) [21]:

(8) $$p_{\theta }\left(z_{0:T}|x_{1:T+1}\right)=p_{\theta }\left(z_{0}|x_{1:T+1}\right)\prod_{t=1}^{T}p_{\theta }\left(z_{t}|z_{t-1},x_{t+1:T+1}\right).$$

This factorization often leads us to approximate the posterior with the variational distributions $q_{\phi }$ of the following form [21]:

(9) $$q_{\phi }\left(z_{0:T}|x_{1:T+1}\right)=q_{\phi }\left(z_{0}|x_{1:T+1}\right)\prod_{t=1}^{T}q_{\phi }\left(z_{t}|z_{t-1},x_{t+1:T+1}\right),$$

in which the parameters of the approximate posterior distribution are denoted by ϕ. Although the above factorization is useful for some purposes [21], the factors comprising the right-hand side of Equation (9) are all conditioned on future information, which may not be desirable in many practical situations. In this paper, we propose a different strategy, in which we collect relevant information from a history of past and current observations and use them as conditioning information for variational distributions. Based on the strategy, the corresponding conditional probabilities become

(10) $$q_{\phi }\left(z_{0:T}|x_{0:T}\right)=q_{\phi }\left(z_{0}|x_{0}\right)\prod_{t=1}^{T}q_{\phi }\left(z_{t}|x_{0:t}\right).$$

In the following Section 2.2.3, we explain how the true posterior distribution can be adequately approximated by using variational inference with the $q_{\phi }$ of the above strategy.

#### 2.2.3. Training Based on Variational Approximation

This subsection describes the training of the parameters θ and ϕ with the variational approximation strategy. As discussed, we approximate the true posterior distribution with the variational distributions in the form of Equation (10). For the factors on the right-hand side of the variational distribution in Equation (10), we use normal distributions with an isotropic covariance matrix structure. That is, we use

(11) $$q_{\phi }\left(z_{t}|x_{0:t}\right)=N\left(z_{t}|\mu \left(x_{0:t}\right),\sigma^{2}I\right),t\in \left\{0,\cdots ,T\right\},$$

where N(z|μ,Σ) denotes the multivariate normal distribution with the mean vector μ and the covariance matrix Σ. For finding the mean parameters of the multivariate Gaussians $q_{\phi }\left(z_{t}|x_{0:t}\right)$, t≥0, we use a Transformer-based auto-regressive model, GPT2 [16]. The mean parameters of $q_{\phi }\left(z_{t}|x_{0:t}\right),t\in \left\{0,\cdots ,T\right\}$ are all obtained from the outputs of the single GPT2 Transformer. In the training process, we find the parameters θ and ϕ simultaneously by maximizing ELBO(θ,ϕ), the variational lower bound given as follows [27]:

(12) $$\begin{array}{ccc} \log p({\bar{x}}_{1:T+1}) & \geq & ELBO(\theta ,\phi )\\ \; & = & \sum_{t=0}^{T}E_{z_{t}∼q_{\phi }\left(z_{t}|x_{0:t}\right)}\left[\log p_{\theta }\left({\bar{x}}_{t+1}|z_{t}\right)\right]\\ \; & \; & -KL(q_{\phi }\left(z_{0}|x_{0}\right)\|p\left(z_{0}\right))\\ \; & \; & -\sum_{t=1}^{T}E_{z_{t-1}∼q_{\phi }\left(z_{t-1}|x_{0:t-1}\right)}\left[KL(q_{\phi }\left(z_{t}|x_{0:t}\right)\|p_{\theta }\left(z_{t}|z_{t-1}\right)\right]. \end{array}$$

**Figure 3:**
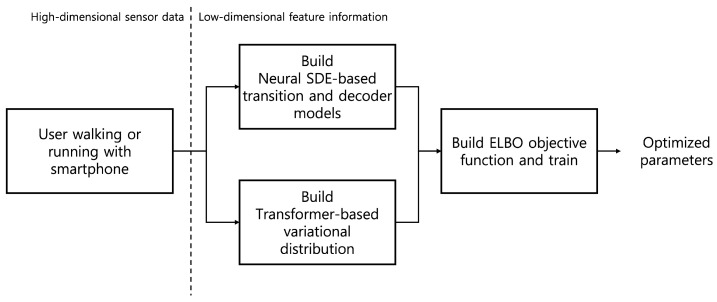
Block diagram for neural SDE and a Transformer-based approach.

The block diagram for our workflow example utilizing neural SDE, GPT2, and ELBO maximization is shown in Figure 3. Overall, the training procedure can be summarized as Algorithm 1. Note that, in the algorithm, we have optional “Contrast Model”-related terms, the meaning of which will be specified in the Section 4.
**Algorithm 1:** Training Procedure1:Prepare Dataset *D*.2:Define Optimizer and hyper-parameters.3:Prepare Generative Model: $p_{\theta }\left(x|z\right)$ and $p_{\theta }\left(z\right)$.4:Prepare Inference Model: $q_{\phi }\left(z|x\right)$.5:(Optional) Prepare Contrast Model: $C_{\omega }\left(x,z\right)$.6:**while** not converged **do**7:    Sample Data Points: x∼D.8:    Sample Latent Points: $z∼q_{\phi }\left(z|x\right)$.9:    Compute Conditional Likelihood $p_{\theta }\left(x|z\right)$ and KL divergence $KL(q_{\phi },p)$.10:    (Optional) Compute Contrastive Loss.11:    Evaluate Total Loss L(x;(θ,ϕ,(optional)ω)).12:    Estimate Monte Carlo Approximations to $\nabla_{\theta }L,\nabla_{\phi }L$, and (optional) $\nabla_{\omega }L$.13:    Update θ,ϕ, and (optional) ω using Optimizer.14:**end while**

## 3. Experiments

**Figure 4:**
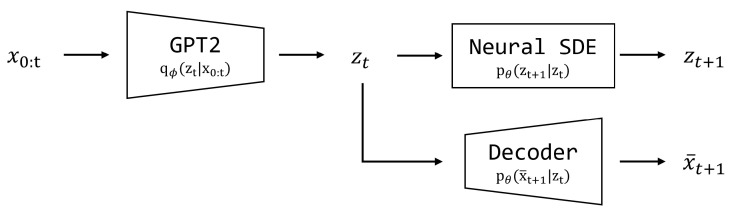
A schematic diagram for the structure of the proposed method: Generative component (neural SDE and MLP decoder) and inference component (GPT2).

In our experiments, we address the problem of characterizing human motions with smartphone sensor data and the proposed algorithm. For the problem formulation, we model the transitions in latent space, decoders, and variational distributions with neural SDEs [14], MLP [20], and GPT2 [16], respectively, and maximize the ELBO resulting from the variational approximation. A schematic diagram for the main components of the proposed method is shown in Figure 4.

For the motions, we considered walking, running, squats, and jumping jacks in this section. We believe the proposed algorithm to be applicable to more types of motions, and we are planning to address its applicability in future follow-up research.

### 3.1. Data Collection

**Figure 5:**
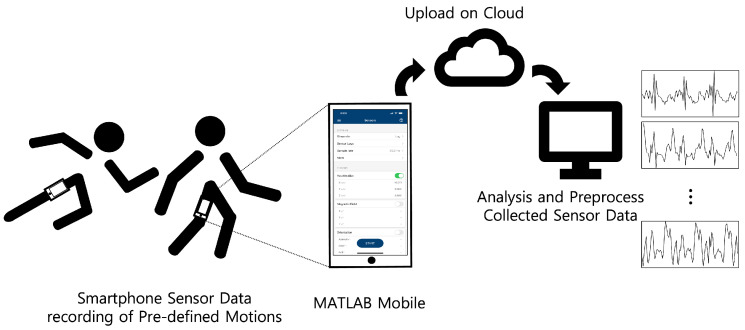
Configuration for acquiring and processing sensor data.

Before training for the proposed method, data collection was conducted, and an overview of procedures for acquiring and processing sensor data is shown in Figure 5. We considered four motions (walking, running, squats, and jumping jacks) for ten subjects. For the motions of walking and running, we collected the data in a straight one-way path at the Korea University R&D Center. The motion data for squats and jumping jacks were collected in our lab. Information on the subjects is provided in Table 1.

**Figure 6:**
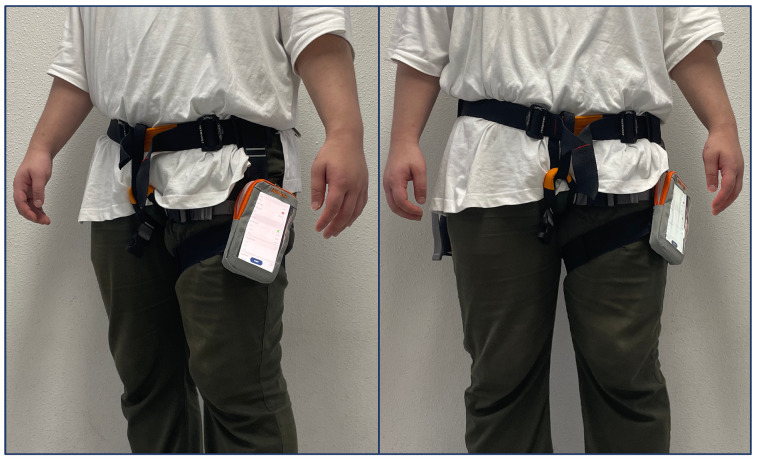
Smartphone location used in the experiments.

For the experiment, we performed the data collection procedures, and then trained a model. First, we utilized the MATLAB Mobile [28] application, which was installed on a smartphone (Apple iPhone XS Max [29]), to obtain gyroscope sensor data. As shown in Figure 6, the smartphone was located on the left side of the leg, which is close to a trousers pocket. In addition, the screen of smartphone was set to face outward. To obtain more information from the sensors, the sampling rate for data collection was set at 30 Hz by increasing the pre-determined default value (10 Hz) on MATLAB Mobile.

The data collection procedure is as follows:

(a) A straight corridor (about 75 m) for walking and running motion and a place unobstructed by people for the other motions (squats and jumping jacks) were chosen.
(b) A smartphone was placed on the left side of the subject’s left leg, in a location similar to the trousers pocket.
(c) MATLAB Mobile was used for accessing built-in sensors on the iPhone with a sampling rate of 30 Hz.
(d) Motions were executed by each subjects.
(e) During step (d), the raw sensor data were collected by the gyroscope sensor.
(f) After acquiring the sensor data, the data were automatically uploaded to a cloud server provided by MathWorks, and the data were accessed via the computer used to train the model.

Second, we conducted preprocessing to input the obtained data into a model and used the deep learning framework, PyTorch [30], to implement and train the model. The acquired raw gyro sensor signals were three-dimensional data, in *x*-, *y*-, and *z*-directions. In addition to the raw sensor signals, we also considered magnitude information, resulting in four-dimensional data. More detailed description of the sensor signals is provided in Table 2. Furthermore, the data were normalized by means of z-scores. Details of the hyper-parameters used in our experiments are provided in the Appendix A.

### 3.2. Experimental Results

In this section, we describe the data details and experimental settings in order to illustrate how the latent trajectories are obtained from the dynamic human motions of walking, running, squats, and jumping jacks. The specific definitions concerning the motions are as follows: Running and walking are distinguished by whether a point exists during the action when both feet are simultaneously off the ground [31]. Squats are a motion in which one stands with legs slightly apart, bends the knees to lower the hips, and then returns to the original position [32]. Jumping jacks are performed by jumping from the attention pose, with the feet spread and hands going overhead, and then returning to the original upright pose from the jump [33]. We collected a sensor dataset from ten subjects at a frequency of 30 Hz, and for each subject, 80% of the data was used as training dataset, and the remaining 20% was used for test datasets. To train the proposed model, we used the AdamW optimizer [34], which is a modification of the widely used Adam optimizer, and is known to improve weight decay. Most notably, the weight decay of the AdamW optimizer can help decrease the chances of overfitting. For the batch size in the training phase, we used B = 128. The observations of the input to the model are four-dimensional, consisting of three normalized gyro outputs along with their normalized magnitude.

**Figure 7:**
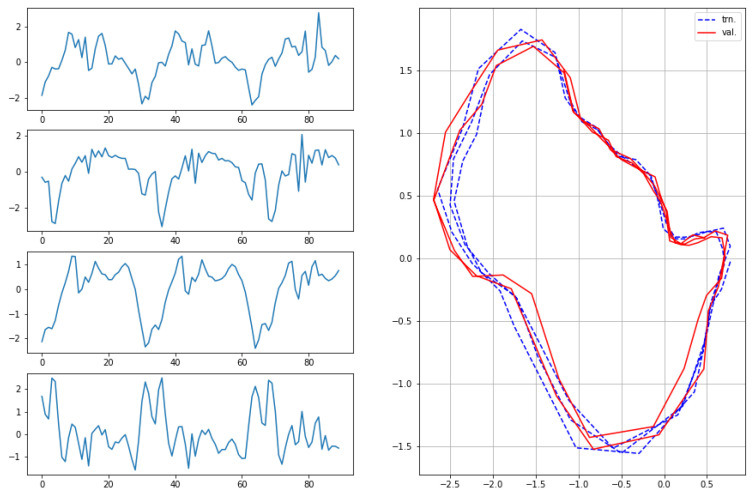
Observations from sensors and corresponding latent sequences for walking.

**Figure 8:**
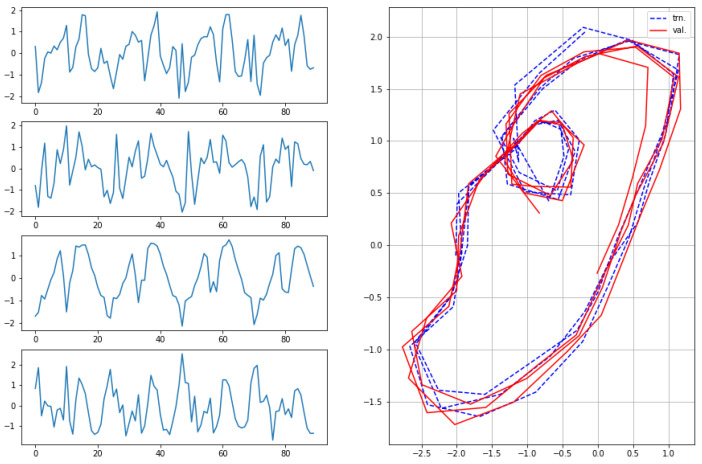
Observations from sensors and corresponding latent sequences for running.

**Figure 9:**
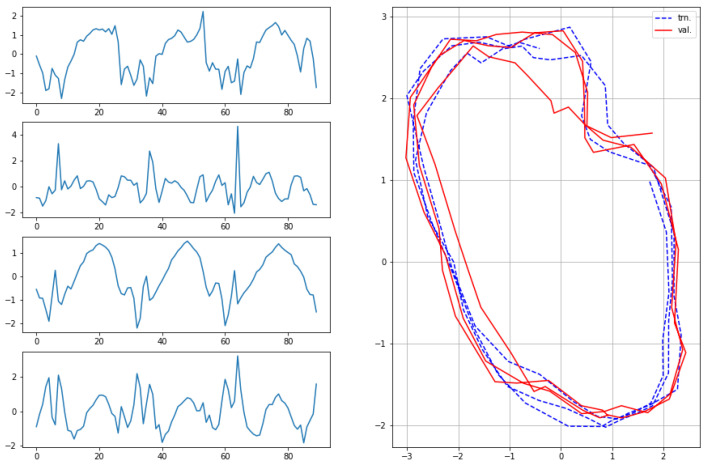
Observations from sensors and corresponding latent sequences for squats.

**Figure 10:**
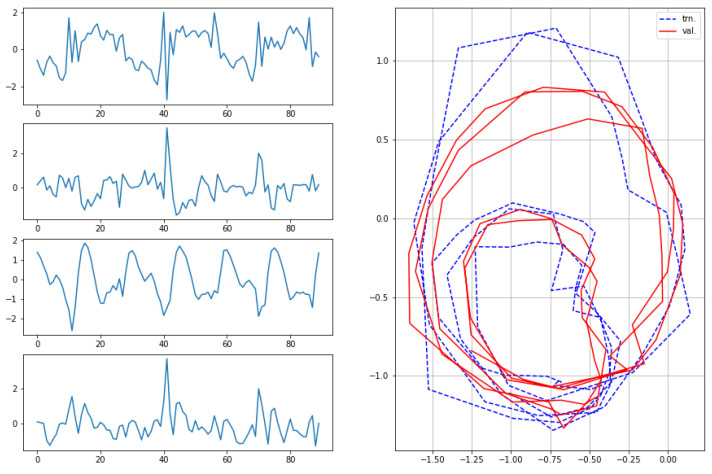
Observations from sensors and corresponding latent sequences for jumping jacks.

**Figure 11:**
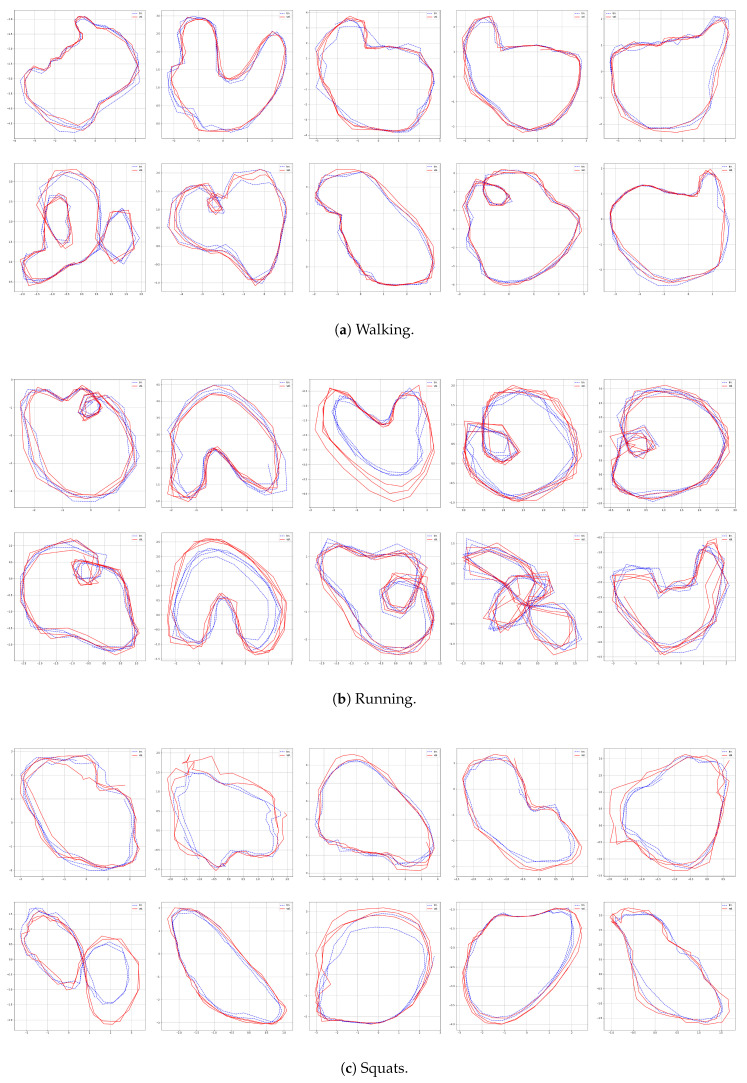

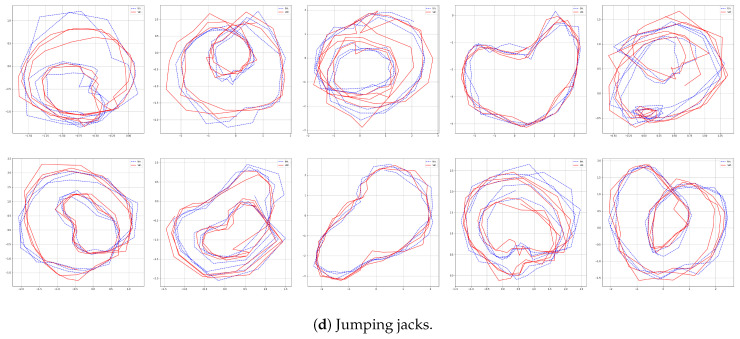
Latent trajectories of walking, running, squats, and jumping jacks for all subjects.

The results for the first subject are shown in Figure 7, Figure 8, Figure 9 and Figure 10, in which we omit a few steps of initial transients. The results of Figure 7, Figure 8, Figure 9 and Figure 10 indicate that the proposed method successfully transformed the high-dimensional sequences of noisy observation data from the smartphone sensors to low-dimensional latent trajectories. For one motion, the latent trajectories of the training and validation data with their common characteristics in fact shared similar patterns in latent space, and were inherently different from other motions, as is shown from their corresponding trajectories in the latent space. All the motions we consider here in this paper contain repetitive sequences. The results of the latent trajectory show that they all contain repetitive components. Furthermore, each motion has a different frequency, e.g., the frequency of walking and running are about 1/30 steps and 1/20 steps, respectively, and the latent trajectories represent these periodic properties. We collected the latent trajectories of each motion for all subjects, and show them in Figure 11.

As mentioned, we considered four motions (walking, running, squats, and jumping jacks) for characterizing motions in the latent space. These motions share some properties, and after performing our characterization process, we obtained the following two interpretations with regard to resultant latent trajectories. (1) Repetitiveness in latent space: The motions of walking, running, squats, and jumping jacks are all repetitive, and accordingly, the resultant latent trajectories show that they all contain repetitive aspects. (2) By comparing the sensor trajectories in the time domain with their corresponding latent trajectories, one can see that these motions have different frequencies in the time domain, while their resultant frequencies in the latent space remain almost the same with their corresponding frequencies. These motions should be interpreted differently from each other, which is clearly shown from their corresponding trajectories in the latent space. This indicates that, in a sense, our latent trajectories acquire distinguishable features while maintaining the original time domain frequencies of their sensor signals.

**Figure 12:**
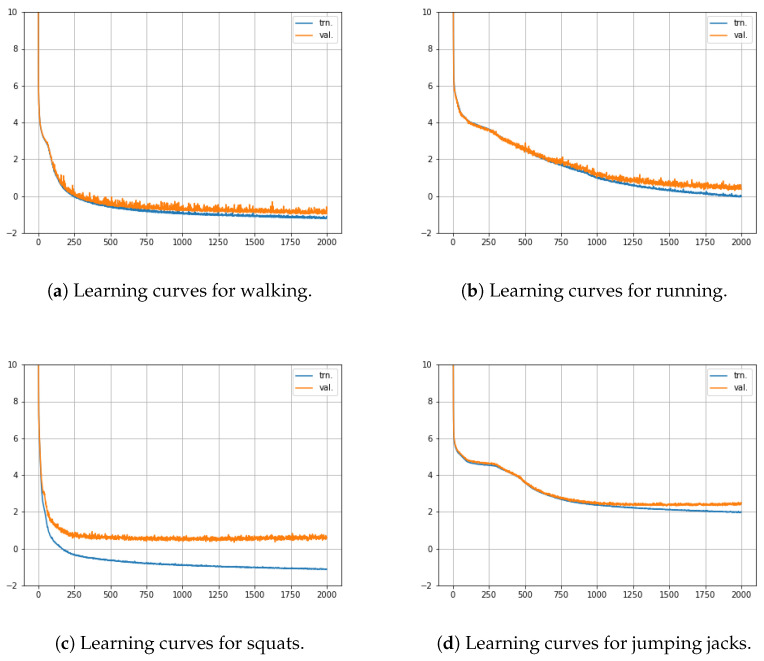
Learning curves for (**a**) walking, (**b**) running, (**c**) squats, and (**d**) jumping jacks.

In addition, we further investigated whether the proposed method is safe from overfitting. In the investigation, we explored the resultant learning curves obtained during the training phase. An exemplar set of learning curves is provided in Figure 12 for the considered set of motions. A usual criterion for overfitting is that the occurrence of overfitting is confirmed when the loss value we seek to minimize for the dataset of validation stops decreasing at some point, and thereafter, the loss value tends to increase. Based on this criterion, one can see that the resultant learning curves of Figure 12 are safe from overfitting.

**Figure 13:**
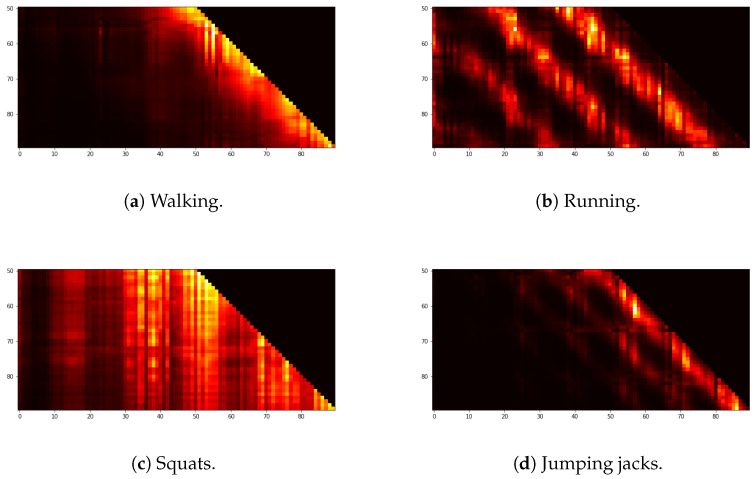
Heatmaps observed in the first embedding layer of the GPT2 Transformer used for the inference network.

The GPT2 Transformer we used for the inference network yielded the feature heat maps as a valuable by-product, which can serve as an explanatory AI capacity. In Equation (4), the attention weight $W_{attn}=\mathrm{softmax}\left(QK^{⊤}/\sqrt{d_{K}}\right)$, where $W_{attn}$ is the size of $R^{T\times T}$ and the sequence length T=90, from self-attention indicates the concentration of temporal information in the inputs. In our experiments, the GPT2 Transformer has two layers, consisting of an attention and feedforward network. The feature heat maps of Figure 13 show some of the resultant attention weights in the first layer of the GPT2 Transformer trained for walking, running, squats, and jumping jacks. The horizontal axis indicates the time steps of the attention weight, which is equal to the sequence length. As for the vertical axis, which indicates the length of the attention layer input, we consider the time span [50,90) to avoid distraction from remote past inputs. They show that running movements are the most inherently periodic during the observed duration.

Finally, we believe that, since the proposed algorithm can characterize repetitive human motions efficiently, it could be used practically in the areas of fitness and healthcare as well as characterizing daily activities of walking and running. For example, one could build a program for exercise and physical fitness, where latent trajectories play the role of guiding into a better motion. In addition, the practical use could be extended further for the general area of customized healthcare services such as personalized healthcare support program and rehabilitation therapy.

## 4. Discussion and Conclusions

### 4.1. Discussion

In this paper, we considered the problem of characterizing dynamic human motions with wearable sensors, specifically, built-in gyroscope sensors on a smartphone. The main rationale for the approach used in this paper is that the high-dimensional sensor signals acquired from the sensors can be represented as lower-dimensional trajectories on a latent space. The main deep learning tool for our characterization is combining neural differential equations and a self-attention model. Since the high-dimensional signals observed in our experiments are originally from intrinsically low-dimensional human motions, and since neural differential equations and self-attention models have recently undergone notable advancement and have been widely applied, the rationale and methods seem reasonable and timely. We proposed a novel approach based on neural SDEs [14], GPT2 [16], and variational approximation [21] to characterize dynamic human motions as determined in a low-dimensional latent feature space. The latent trajectories we obtained by means of the proposed method turn out to be sufficiently unique for each considered subject. Thus, our proposed method can be valuable for distinguishing people from their motion data as measured by smartphone sensors. We will further discuss some related topics in the following section.

#### 4.1.1. User Identification

As mentioned, the main goal of the proposed method is characterizing human motions of walking, running, squats, and jumping jacks from smartphone sensor signals. In this subsection, we consider the problem of extending the main goal of characterizing human motion to enhancing user identification ability by learning latent variables so that they should carry more individually salient features. As a tool for achieving this extended goal, an additional loss term is introduced, which can reflect contrasts among users. Thus, to distinguish the latent variables of different users, a contrast loss term compares the latent variables of different users. The exact definition of the contrast loss term, $C_{\omega ,\phi }$, is as follows:

(13) $$\begin{array}{c} C_{\omega ,\phi }=\\ -\sum_{a,b\neq a}\sum_{t}[E_{z^{\left(a\right)}∼q_{\phi }\left(z^{\left(a\right)}|x^{\left(a\right)}\right)}\left[\log(\sigma \left(C_{\omega }\left(x_{t}^{\left(a\right)},z_{t}^{\left(a\right)}\right)\right)\right]\\ \;+E_{z^{\left(b\right)}∼q_{\phi }\left(z^{\left(b\right)}|x^{\left(b\right)}\right)}\left[\log(\sigma \left(1-C_{\omega }\left(x_{t}^{\left(a\right)},z_{t}^{\left(b\right)}\right)\right))\right]]. \end{array}$$

**Figure 14:**
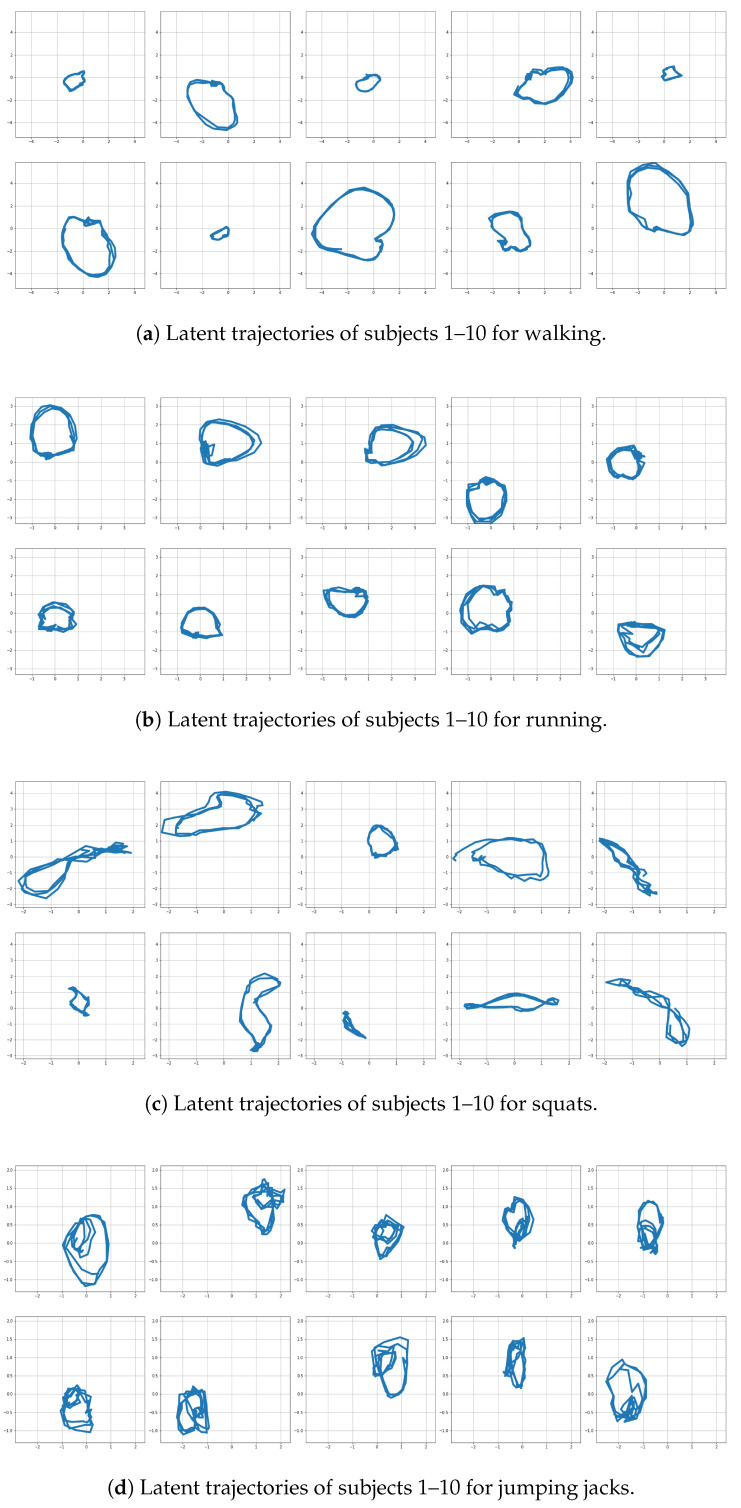
Training results when the additional contrast loss term is included for user identification ability.

In this equation, (a) in the superscript means that the term is defined for user *a*. With the above defined contrast loss term minimized, one can expect that the resultant network can better distinguish latent variables from different users. This contrast network is implemented by as an MLP [20], and the parameter ω represents the weights of the network computing the contrast term. To ascertain the effects of the contrast term, we perform simulations in which training is conducted with the additional contrast loss term included. The training results for the four motions are reported in Figure 14, which show that users’ corresponding latent sequences are indeed distinguishable from each other. These results can serve as a different type of signature that can characterize users by their individual motion patterns.

#### 4.1.2. Optimized Initial Latent States

**Figure 15:**
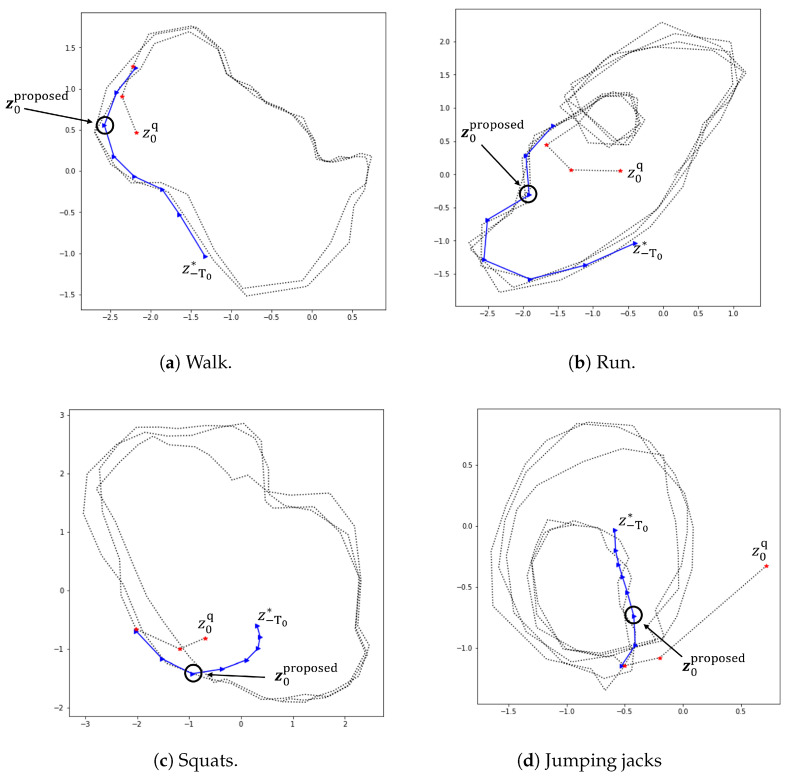
Initial latent state estimation with (blue colored) and without (red colored) optimization over $z_{-T_{0}}^{*}$. $z_{0}^{proposed}$ is the initial state estimation obtained by the proposed optimization method, while $z_{0}^{q}$ is the result following the procedure of Section 2. Note that black dotted lines are the latent trajectories obtained for a test data set.

In this subsection, the problem of how to find the initial latent state more accurately is dealt with. As mentioned in Section 2, the starting point of the latent sequence, $z_{0}$, was obtained by a performing principal component analysis (PCA) with $x_{-m:0}$ as the inputs, where *m* is a small non-negative integer. Since an initial latent state cannot be directly observed, using a dimension reduction technique like PCA is more or less a trade-off needed for normal situations. This section addresses cases in which we have relatively more abundant observations for estimating initial latent states. For related work on data assimilation, one may refer to [35], where the authors study how to obtain the latent initial conditions of a dynamical system under incomplete information. We proceed similarly to find the initial latent state more accurately. More specifically, given the observations $x_{-T_{0}:0}$ from time $-T_{0}$ up to the initial time 0, we make use of the strategy of inferring the latent state that can best reproduce an observed time series. Here, we assume that observations for negative time steps $\left\{-T_{0},\cdots ,-1\right\}$ are available as extra data for estimating initial latent states. This strategy is accomplished by minimizing the discrepancy between the observations $x_{-T_{0}:0}$ and their estimated values via a gradient descent method to find the best $z_{-T_{0}}^{*}$. Figure 15 shows that the optimized initial latent state relying on $z_{-T_{0}}^{*}$ can be placed near the normal latent trajectories, whereas the results of the PCA initialization stay away from the trajectories during a few initial steps.

#### 4.1.3. Other Related Topics: Characterizing Multiple Motions, Normal Latent Region, and Motion Switching

**Figure 16:**
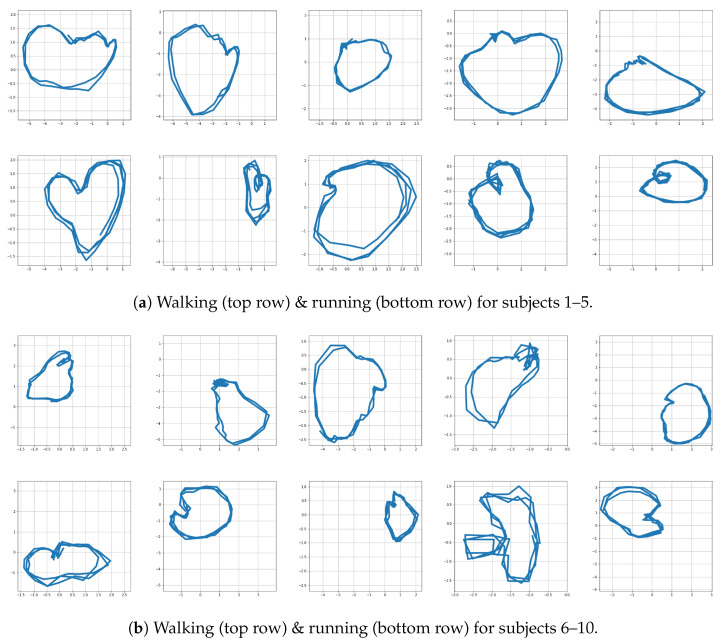
Motions represented in shared latent space $R^{2}$.

In this subsection, we consider the tasks of extending the proposed method for characterizing multiple motions, normal latent region, and motion switching. Since characterizing general motion switching can be challenging, we deal with the extension with focus on walking and running. Covering multiple motions in the formulation for latent trajectories may yield additional flexibility [36]. For this task, multiple trajectories of walking and running were trained together, with the aim of characterizing them on the same shared latent space. When handling multiple motions, we simply collect all the motion data and conduct pre-processing to acquire z-scores for all motion types. Then, instead of learning the weights of the proposed network separately for each type of motion, we train the network with all the data. Since our network is equipped with neural SDEs and the GPT2 as powerful transition and inference components, the trained network is expected to efficiently handle the observations from multiple motions. Furthermore, by using an additional contrast loss term in training, we increase its capacity to learn multiple motions with an enhanced ability for distinguishing different motions. Figure 16 shows the latent trajectories for walking and running motions resulting from the use of a common latent space for these motions. One can see in the figure that, in the latent space, walking is characterized by a low frequency, while running exhibits a higher frequency.

Once the training stage is completed, we can find the latent regions for considered motions based on the training results. For the task of finding the latent region for walking and/or running, we use a straightforward extension of the proposed approach via a kernel density estimation (e.g., [37]). More specifically, we simply collect some latent patterns that appeared during the training stage, and use them for conducting non-parametric density estimation for each motion via a tool of scipy [38].

**Figure 17:**
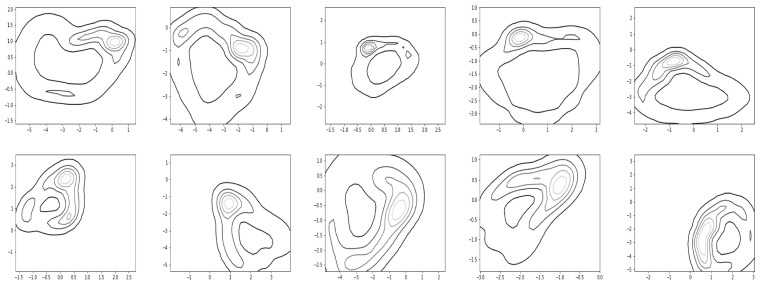
Latent regions found by kernel-based density estimation for walking.

**Figure 18:**
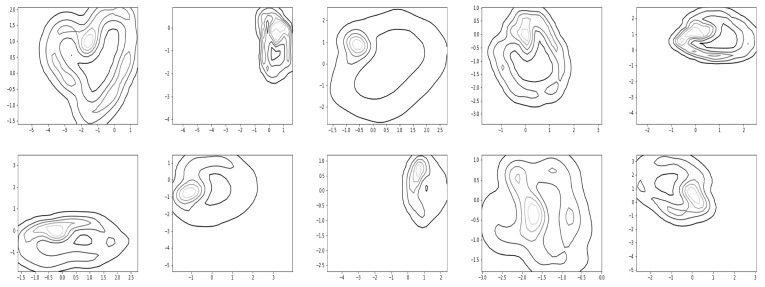
Latent regions found by kernel-based density estimation for running.

Figure 17 and Figure 18 show the resultant density contours of the latent patterns for walking and running, respectively.

**Figure 19:**
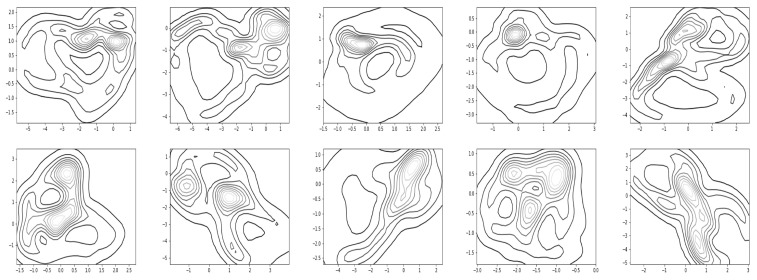
Normal latent regions found by kernel-based density estimation for the data from walking and running.

The capacity about whether a given data point belongs to the normal latent region (see Figure 19), and when a significant deviation or an abnormal trajectory occurs, can issue an alarm to users. With this capacity in mind, we find a distribution for normal latent patterns which are derived from the union of walking and running observation data. Figure 19 shows how relevant contours for the normal latent patterns in $R^{2}$ appeared in the experiments. Since the trajectory deviating from the normal latent region can be quickly noticed, this capacity may be utilized for detecting motion changes. For the task of showing the contours of the density, we utilized a readily available matplotlib function, matplotlib.pyplot.contour [39]).

**Figure 20:**
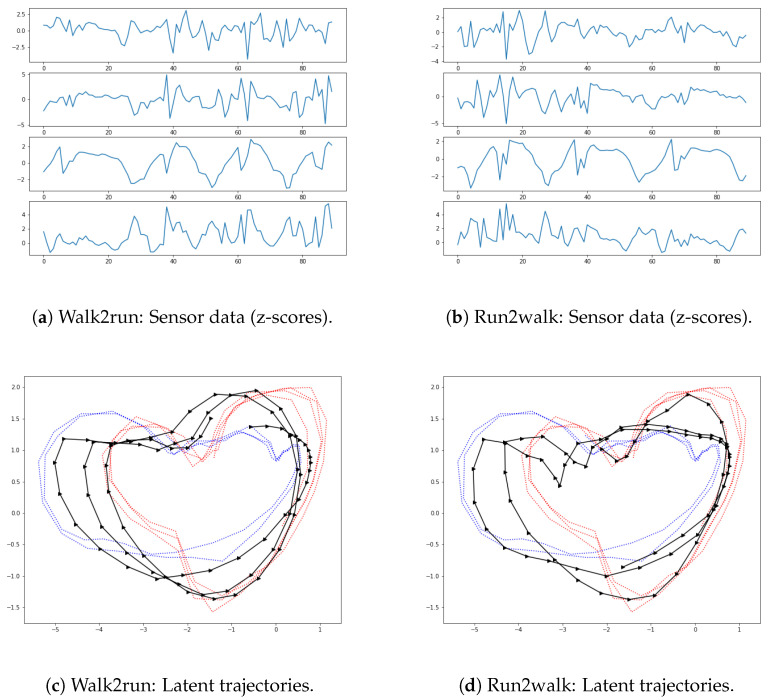
Motion switching represented in observation and latent space. Sub-figures (**c**,**d**) show motion switching (black solid lines) together with latent trajectories of walking (blue dotted lines) and running (red dotted lines).

Finally, we conducted simulations to check whether the resultant model covers characterizing motion switching. First, we trained the network with multiple demonstrations of walking and running. In the test stage, the motions of the first subject were intentionally switched from walking to running, or from running to walking, and we obtained corresponding trajectories following the proposed framework (see Figure 20). The bottom row of Figure 20 shows motion switching in the latent space, where it computed the moving average of the latent trajectories with the rolling window size of five steps. Overall, the results of Figure 16, Figure 17, Figure 18, Figure 19 and Figure 20 show that our framework can also cover the problem of handling multiple motions and motion switching.

### 4.2. Conclusions

In this paper, we investigated the problem of smartphone sensor-based human motion characterization with neural stochastic differential equations and a transformer model. We utilized built-in gyroscope sensors of a single iPhone XS Max unit tied on near the left trousers pocket. From the unit sensors, we obtained the angular velocities along the *x*-, *y*-, and *z*-axes, and computed their total magnitude. We normalized the xyz angular velocities and the magnitude, respectively, and utilized them as our input features. The human motions involved in our investigations include walking, running, squats, and jumping jacks. For the characterization of the motions, we proposed a novel approach consisting of neural SDE-based latent dynamics modeling and GPT2-based variational approximation.

The novelty of the proposed approach can be summarized as follows: Networks for sequential inference are often implemented with accumulated observations from the present and future. In contrast to such inference models, ours is based on a history of past and current observations for variational distribution, which should be practical in applications. Our inference model uses GPT2, which is more advanced than conventional recurrent network-based models. Our approach makes use of transforming the sequences of high-dimensional observations into a latent space along with decoding for the average of reconstruction and prediction. The dimensionality of the latent space is 2, which is a convenient choice for characterization and visualization. The results shown in two-dimensional latent space are capable of efficiently capturing the characteristics of users’ dynamic motion patterns. We formulated the transition of the latent generative component with neural SDE, which can handle stochastic dynamical features in the latent space. We also considered an optimization for obtaining more accurate initial latent state when relevant observations are available for the optimization. We presented further related discussion on how to enhance user identification ability by learning latent variables so that they should carry more individually salient features. For the enhancement, an additional loss term capable of reflecting contrast among users was introduced. The results when the contrastive loss term was added showed the differences in user-specific patterns more clearly in the latent space. After addressing how to obtain a latent region for normal motions, we also discussed how to store multiple motions in the latent space, and how to find motion switching among multiple motions.

One of the important issues that should be addressed in future studies concerns the practical possibility of implementing the proposed method in current smartphone systems. We believe that, since the proposed method addresses the practical needs like motion characterization in a latent space and user identification, deploying the trained networks into a smartphone would have much practical value. We also believe that its implementation and operation in real-time are all possible. Important related works remain to be conducted on further aspects such as comparison studies and more extensive experiments. We believe that they will uncover strengths and weaknesses of the proposed approach more clearly, and enable further refinements of multiple aspects of this approach. Examining different types of data structures and different types of human motions are important topics, for which more research is needed, in light of the important applications of this area of research for health care, fitness, and user-device interaction.

## Figures and Tables

**Table 1:** Profiles of the recruited subjects.

Subjects	Gender	Height (cm)	Weight (kg)
Subject 1	M	175	80
Subject 2	M	172	67
Subject 3	F	163	68
Subject 4	M	164	62
Subject 5	M	188	75
Subject 6	M	174	65
Subject 7	M	167	56
Subject 8	M	171	74
Subject 9	F	164	70
Subject 10	F	158	57
Average		169.6	67.4

**Table 2:** Smartphone unit’s feature data set.

Notation	Meaning
$\omega_{x},\omega_{y},\omega_{z}$	Angular velocities around the x,y,z-directions, respectively
$\omega_{T}$	Square root of the sum of squares of angular velocities, $\sqrt{\omega_{x}^{2}+\omega_{y}^{2}+\omega_{z}^{2}}$

## Data Availability

Not applicable.

## Appendix A. Details about Architecture and Hyper-Parameters

As mentioned, we used a small GPT2 structure consisting of two layers, a single head and an embedding size of 16, which turned out to be sufficient for our purposes. We found that, when the embedding size is further increased, the resultant performance worsens. Since the main concern of this paper is smartphone or mobile applications, considering small sizes for the structure should suffice. The detailed architecture of the GPT2 Transformer used for the study is shown in Figure 2a. In addition, the detailed structure of the neural SDE used for the study is provided in Figure 2b. For the drift network of the neural SDE, we used MLP with two hidden layers. Each hidden layer consists of a total of 32 hidden units with LipSwish activation functions. We have chosen 32 hidden units and the LipSwish activation function, following the practice of [14]. In this paper, the decoder network is a two-layer MLP network with H = 256 hidden nodes in each layer. Note that it is a slightly smaller size compared to those typically used in large-scale Transformers. Again, since we are concerned with smartphone or mobile applications, choosing H = 256 should be sufficient. Finally, a sequence length of T = 90 has been chosen so that the sequence includes several periods of considered repetitive motions.

**Table A1:** Hyper parameters.

Name	Symbol	Value
Batch size	*B*	128
Sequence length	*T*	90
Embedding size	*E*	16
Latent dimensions	*k*	2
GPT2 number of layers	–	2
GPT2 number of heads	–	1
Drift MLP sizes	–	[32,32]
Initial diffusion std	$\sigma_{\theta }$	0.2
Initial state std	$\sigma_{0}$	0.2
Decoder MLP sizes	–	[256,256]
Learning rate	λ	0.001
KL loss scale	β	1.0
Contrast loss scale	γ	100.0
